# Beyond AI readiness: an information-integrity readiness framework for governing synthetic media in Somalia

**DOI:** 10.3389/frai.2026.1959660

**Published:** 2026-10-27

**Authors:** Abdinor Abukar Ahmed, Abshir Abdisheekh Mohamed, Mohamed Khalif Ali, Yasin Haji Mohamed, Mohamed Aweis Omar, Abdirahman Ali Mohamed, Ismail Mahad Hersi

**Affiliations:** 1 Faculty of Engineering and Computer Technology, Somali International University, Mogadishu, Somalia; 2 Benaadir Research, Consultancy and Evaluation Centre (BRCE), Mogadishu, Somalia; 3 Somali National University (SNU), Mogadishu, Somalia

**Keywords:** AI governance, artificial intelligence readiness, Deepfakes, digital resilience, fragile states, information integrity, Somalia, synthetic media

## Abstract

Generative artificial intelligence has transformed synthetic media from a specialized technical concern into a systemic governance challenge. Existing AI readiness frameworks evaluate whether states can adopt, deploy, and regulate AI, but they do not systematically assess whether societies can preserve the authenticity, trustworthiness, and resilience of information circulating within AI-enabled environments. This Perspective introduces Information-Integrity Readiness (IIR) as a distinct but complementary governance construct. Through an interdisciplinary narrative synthesis of AI governance, readiness, digital resilience, misinformation, and synthetic-media scholarship, the article identifies an Information-Integrity Gap and develops a five-pillar framework comprising institutional readiness, technical authenticity readiness, legal and regulatory readiness, societal resilience readiness, and sector-specific readiness. The framework is theoretically anchored in institutional theory, policy capacity theory, and socio-technical systems theory, which, respectively, explain legitimacy and coordination, the analytical-operational-political capacities required for implementation, and the interdependence of technical and social governance arrangements. Somalia is used as an illustrative application, while Kenya is included only as a concise transferability illustration rather than a second comparative case. The Perspective advances four theory-linked propositions for future empirical testing, clarifies qualitative maturity criteria for illustrative readiness assessment, outlines candidate indicators for future operationalization, and proposes a phased policy roadmap. The central argument is that the next generation of AI governance must move beyond measuring technological capability toward evaluating whether societies can sustain trustworthy information ecosystems under conditions of pervasive synthetic media.

## 1 Introduction

Generative AI has dramatically reduced the cost of producing highly convincing text, images, audio, and video, enabling synthetic content to be created and disseminated at unprecedented scale and speed (Ferrara, 2026; Waditwar, 2025). Amplified through algorithm-driven social media platforms, these synthetic artifacts increasingly shape political discourse, public opinion, and institutional trust by exploiting both algorithmic systems and human cognitive vulnerabilities (Aditya, 2025; Romanishyn et al., 2025). The resulting challenge extends well beyond misinformation. Synthetic media undermines the long-standing assumption that audiovisual content provides reliable evidence of reality, threatening the credibility of journalism, science, governance, and democratic decision-making (Godulla and Hoffmann, 2025). As synthetic content, identities, and interactions become progressively more difficult to distinguish from authentic communication, societies face what has been described as the Generative AI Paradox: the widespread availability of synthetic media may ultimately reduce confidence in digital evidence itself, increasing the institutional and societal cost of establishing truth (Ferrara, 2026).

This transformation redirects attention from AI adoption alone toward the governance capacity required to preserve trustworthy information ecosystems. Information integrity refers to the authenticity, trustworthiness, and resilience of information throughout its lifecycle, encompassing its creation, modification, dissemination, and verification (Ghiurău and Popescu, 2025). Unlike misinformation research, which primarily evaluates content accuracy, or cybersecurity, which focuses on protecting digital systems, information integrity concerns the institutional and technical arrangements that preserve provenance, accountability, and verifiable authenticity (Gregory, 2022). Concepts such as authenticity infrastructure and authenticity debt further illustrate that maintaining trustworthy information increasingly depends on governance mechanisms capable of documenting media provenance, supporting verification, and sustaining public confidence as synthetic content becomes commonplace (Ferrara, 2026; Gregory, 2022).

Despite rapid advances in AI governance, existing readiness frameworks remain primarily designed to evaluate countries’ capacity to develop, adopt, and regulate artificial intelligence. These frameworks emphasize technological infrastructure, innovation capability, digital skills, regulatory maturity, and institutional preparedness (Holmström, 2022; Ouakil et al., 2025). Global readiness indices and public-sector AI governance models have substantially advanced comparative assessment, yet they provide limited coverage of many African countries, including Somalia, while inadequately reflecting governance realities in fragile and developing digital states (Ayana et al., 2024; Diallo et al., 2025; Plantinga et al., 2024). More fundamentally, their central unit of assessment is AI capability and governance; recent synthetic-media work, including detection-oriented approaches, addresses important harms but does not by itself provide a national readiness architecture for preserving information authenticity across institutions, law, society, and sectors (Moyo et al., 2026).

This distinction represents a shift in the object of governance. AI readiness asks whether governments and institutions can responsibly develop, deploy, and regulate AI systems. Synthetic media creates an additional question: whether states can preserve the evidentiary and institutional conditions under which information can still be trusted. That second question cannot be answered by infrastructure, innovation, or regulatory maturity alone. It requires coordinated capacity to authenticate media, assign responsibility, manage incidents, communicate uncertainty, protect rights, and sustain public confidence across sectors.

Somalia provides an illustrative context for examining this emerging governance challenge. Rapid expansion of mobile connectivity, digital financial services, and internet access has accelerated the country’s digital transformation, while institutional safeguards for protecting information authenticity remain comparatively underdeveloped (Bengio et al., 2024; Motwani and Sahoo, 2026). Rather than presenting Somalia as an exceptional crisis context, this Perspective employs the country as an illustrative case through which the growing divergence between digital adoption and governance capacity becomes particularly visible. Somalia’s exclusion from major global AI readiness assessments further demonstrates how fragile states frequently remain outside prevailing evaluation frameworks despite facing heightened vulnerability to synthetic media harms (Diallo et al., 2025).

This Perspective addresses a narrower problem than AI readiness: national preparedness to preserve authentic, trustworthy, and resilient information ecosystems when synthetic media becomes pervasive. An AI-driven conceptual framework centered on detecting fake-news and deepfake content (Moyo et al., 2026) addresses an important part of the problem; in IIR, detection is treated as one technical capability within a broader readiness architecture that also requires institutional ownership, enforceable accountability, societal resilience, and sector-specific operationalization. Multistakeholder governance approaches and policy recommendations for democratic resilience (Romanishyn et al., 2025) similarly address important components, but not the integrated readiness question posed here. We therefore introduce Information-Integrity Readiness (IIR) as a distinct governance construct that complements rather than replaces existing AI readiness and synthetic-media governance approaches.

To explain why information-integrity capacity varies across states and why isolated technical interventions are insufficient, this Perspective draws on three complementary theoretical lenses. Institutional theory directs attention to the legitimacy, stability, coordination, and enforcement of governance arrangements (Scott, 2010; Wang and Ching, 2013). Policy capacity theory distinguishes the analytical capacity to diagnose problems and evaluate responses, the operational capacity to mobilize organizations and resources, and the political capacity to secure legitimacy, coordination, and sustained support (Wu et al., 2015; Howlett, 2016). Socio-technical systems perspectives explain why information integrity is produced through interactions among technologies, institutions, rules, professional practices, platforms, and users rather than through technical detection alone (Sanfilippo et al., 2025; de Gennaro et al., 2025). These perspectives provide the theoretical mechanisms through which the five IIR pillars are interpreted and connected.

The article makes five contributions. First, it defines a distinct object of readiness—the national information ecosystem—rather than proposing another AI-adoption or content-detection model. Second, it identifies the Information-Integrity Gap that arises when AI readiness frameworks govern systems but leave the integrity of information environments insufficiently assessed. Third, it provides an explicit theoretical foundation by integrating institutional theory, policy capacity theory, and socio-technical systems theory. Fourth, it organizes existing but dispersed governance capacities into five interdependent and partially non-substitutable pillars, with qualitative maturity criteria and theory-linked propositions that create a pathway toward future measurement; the novelty therefore lies in the integrated readiness architecture rather than in claiming that each pillar is individually new. Fifth, using Somalia as an illustrative application and Kenya as a concise transferability illustration, it demonstrates the diagnostic and potentially transferable value of IIR for fragile and developing digital states.

## 2 Methodology

This Perspective adopts an interdisciplinary narrative literature synthesis to develop Information-Integrity Readiness (IIR) as a conceptual governance framework. A conceptual Perspective is appropriate because the objective is not to generate new empirical evidence, but to integrate fragmented scholarship into a coherent analytical construct capable of addressing an emerging governance challenge (Şenyapar, 2026; Tim and Leidner, 2023). Unlike systematic reviews, which primarily synthesize empirical findings to answer predefined research questions, narrative conceptual synthesis is particularly suited to theory development in rapidly evolving multidisciplinary fields where knowledge remains dispersed and conceptual boundaries are still emerging (Undheim, 2024; Zuiderwijk et al., 2021).

Literature identification was purposive and concept-driven rather than systematic. The synthesis drew on peer-reviewed journal literature, major governance and policy scholarship, and relevant national or international policy documents located through multidisciplinary academic search sources and reference chaining. The literature considered primarily covered the period from 2021 to 2026, while earlier foundational theoretical works were retained where necessary to establish institutional, policy-capacity, and socio-technical mechanisms. Search concepts were organized around combinations of “artificial intelligence readiness,” “AI governance,” “synthetic media,” “deepfakes,” “information integrity,” “digital resilience,” “digital trust,” “misinformation governance,” “authentication,” “provenance,” “institutional capacity,” “policy capacity,” and “socio-technical systems.” Source selection was guided by conceptual relevance: sources were retained when they directly informed national or institutional preparedness, authenticity and provenance, regulatory or implementation capacity, societal resilience, or sector-specific responses to AI-enabled information risks. Sources concerned only with technical model performance, without a governance or information-integrity connection, were not central to the conceptual synthesis. Because this was a narrative Perspective rather than a systematic review, no claim of exhaustive retrieval or formal study-quality scoring is made.

The framework was developed through the iterative synthesis of four complementary bodies of literature. First, AI governance scholarship was examined to identify how governments organize institutional, regulatory, and policy responses to artificial intelligence (Batool et al., 2025; Zuiderwijk et al., 2021). Second, national and organizational AI readiness frameworks were reviewed to determine the governance dimensions currently used to evaluate preparedness and to identify their limitations, particularly within fragile and developing digital states (Chounta et al., 2024; Tsakalerou et al., 2025). Third, literature on synthetic media and information integrity was synthesized to examine how generative AI challenges authenticity, institutional trust, provenance, and digital governance (Godulla and Hoffmann, 2025; Whittaker et al., 2023). Finally, policy documents and national AI strategy analyses were reviewed to understand how governments operationalize AI governance across different institutional settings (Bareis and Katzenbach, 2022; Guenduez and Mettler, 2023). Collectively, these literature streams established the conceptual foundation for identifying governance capacities insufficiently represented within existing readiness frameworks.

The analytical process proceeded in four stages. The first stage identified the dominant object of assessment in existing readiness frameworks: the capability to adopt, deploy, and regulate AI. The second stage mapped synthetic-media risks that are not adequately captured by that object, including provenance failure, identity deception, evidentiary uncertainty, institutional coordination problems, sector-specific harms, and declining public confidence. The third stage extracted and clustered recurring governance capacities across the literature. Coordination, institutional ownership, accountability, incident command, and resourcing were grouped as institutional readiness; authentication, provenance, forensic verification, interoperability, and trusted identity mechanisms as technical authenticity readiness; enforceable duties, platform obligations, remedies, evidentiary rules, and rights safeguards as legal and regulatory readiness; literacy, awareness, trusted communication, inclusion, and interpretive capability as societal resilience readiness; and domain-specific protocols, exercises, escalation arrangements, and continuity mechanisms as sector-specific readiness. To reduce arbitrary overlap, concepts were assigned according to their primary governance function while cross-pillar dependencies were retained in the theoretical interpretation. The fourth stage checked the resulting structure for a distinct object of readiness, internal coherence, functional non-substitutability, transferability across settings, and potential operationalization through future indicators.

The proposed IIR framework therefore emerged through inductive conceptual synthesis rather than the creation of entirely new governance dimensions. Existing sociotechnical governance models, digital resilience theory, multidimensional resilience frameworks, institutional-capacity scholarship, and policy-capacity theory provided complementary analytical foundations for organizing dispersed concepts into a unified governance construct (Domorenok et al., 2021; Sanfilippo et al., 2025; Tim and Leidner, 2023; Wu et al., 2015). The resulting five readiness pillars—institutional, technical authenticity, legal and regulatory, societal resilience, and sector-specific readiness—represent recurring governance capacities identified across the synthesized literature rather than normative categories developed independently (Risnanto et al., 2025; Rød et al., 2025; Tsakalerou et al., 2025).

The five-pillar structure was retained because each pillar performs a non-substitutable governance function. Institutional capacity establishes legitimate ownership and coordination; technical capacity makes authenticity claims verifiable; legal capacity defines enforceable duties and remedies; societal capacity enables informed interpretation and resilience; and sector-specific capacity translates general safeguards into operational procedures. The pillars are therefore presented as a minimum complete set rather than an exhaustive catalogue of policy interventions. This logic is consistent with policy-capacity scholarship, which distinguishes analytical, operational, and political competences and emphasizes that capacity depends on resources and capabilities across individual, organizational, and system levels (Wu et al., 2015), and with socio-technical approaches that treat meaningful technological outcomes as products of interaction among technical, human, organizational, and institutional elements (Racherla and Mandviwalla, 2013).

Somalia is employed as an illustrative application rather than an empirical case study. As a fragile yet rapidly digitizing state, Somalia demonstrates how expanding digital transformation can coexist with institutional constraints, fragmented governance, uneven regulatory capacity, and limited interoperability (Risnanto et al., 2025). The country application was interpreted using available secondary evidence and the qualitative maturity criteria defined later in the manuscript; the purpose is diagnostic illustration rather than national ranking or causal inference.

This approach has important boundaries. It does not claim that the five pillars have been empirically validated, that the literature search was exhaustive, or that the Somalia classifications constitute statistically derived measurements. The purpose is theory development and diagnostic illustration. Future work should test construct validity, develop and weight indicators, undertake expert validation, and compare countries and sectors using primary and secondary evidence.

## 3 AI readiness frameworks and the information-integrity gap

The concept of AI readiness has evolved from an organizational assessment tool into a multidimensional governance framework for evaluating how governments and institutions prepare for the adoption, deployment, and regulation of artificial intelligence. Contemporary readiness frameworks increasingly integrate technological infrastructure, institutional capacity, human capital, ethical governance, regulatory maturity, and digital innovation as complementary dimensions of responsible AI development. This evolution reflects the recognition that successful AI implementation depends not only on technological capability but also on governance systems capable of ensuring accountability, transparency, and responsible deployment (Holmström, 2022; Kurhayadi, 2025).

International governance initiatives—including the OECD AI Principles, the UNESCO Recommendation on the Ethics of AI, and national frameworks such as Singapore’s Model AI Governance Framework—have further expanded this perspective by incorporating legal, ethical, and societal considerations into AI governance (Plantinga et al., 2024; Taeihagh, 2021). Sector-specific adaptations have similarly strengthened governance maturity by introducing oversight mechanisms, algorithm evaluation, implementation guidance, and continuous monitoring across domains such as healthcare (Hussein et al., 2026; Wirtz et al., 2022). Together, these frameworks have significantly advanced governments’ ability to evaluate AI preparedness, identify implementation priorities, and strengthen institutional governance capacity (Hussein et al., 2026). Evidence from African AI readiness assessments likewise suggests that stronger governance capacity contributes to improved government effectiveness and control of corruption (Ouakil et al., 2025).

Despite these advances, existing readiness frameworks remain conceptually oriented toward governing AI systems rather than governing information ecosystems. Their principal objective is to evaluate whether countries possess the institutional and technological capacity to adopt, regulate, and responsibly deploy artificial intelligence. Consequently, readiness is predominantly interpreted through innovation capability, infrastructure, regulatory maturity, digital skills, and organizational preparedness. These dimensions remain essential, but they do not determine whether information can be authenticated, whether provenance can be verified, whether synthetic-media incidents can be coordinated across institutions, or whether citizens can continue to rely on digital evidence once synthetic media becomes pervasive.

This limitation has become increasingly significant as generative AI transforms digital information environments. Synthetic media enables highly convincing fabricated text, audio, images, and video to be produced at negligible cost and rapidly amplified through engagement-driven digital platforms (Waditwar, 2025). These capabilities extend beyond political misinformation to facilitate identity deception, financial fraud, health misinformation, reputational manipulation, and coordinated influence operations that directly challenge institutional trust and public safety (Romanishyn et al., 2025). The governance challenge therefore extends beyond regulating AI technologies themselves to safeguarding the integrity of the information ecosystems within which those technologies operate (McCarthy et al., 2023; Romanishyn et al., 2025; Waditwar, 2025).

Three interrelated limitations explain why existing AI readiness frameworks remain insufficient for this emerging governance challenge. First, many frameworks implicitly assume levels of institutional maturity, technical expertise, and financial resources that remain difficult to achieve in fragile and developing digital states (Hussein et al., 2026; Taeihagh, 2021). Second, they predominantly emphasize governance principles—including transparency, ethics, accountability, and responsible AI—while providing comparatively limited guidance for operationalizing these principles within resource-constrained institutional environments (Taeihagh, 2021). Third, and most fundamentally, they were developed to evaluate readiness for AI governance rather than readiness to preserve authentic information under conditions of large-scale synthetic content generation.

This distinction reveals what we conceptualize as the Information-Integrity Gap. Existing frameworks acknowledge misinformation, cybersecurity, or digital trust as individual governance concerns (Wirtz et al., 2022). Within the literature synthesized for this Perspective, these concerns are generally treated as separate functions rather than as one national readiness architecture that jointly assesses institutional, technical, legal, societal, and sector-specific capacity to authenticate information, establish media provenance, coordinate governance responses, strengthen accountability, and sustain resilience to synthetic-media manipulation. The claim is therefore not that existing scholarship ignores information integrity, but that the reviewed approaches do not organize these capacities around a dedicated system-level readiness construct.

Evidence across multiple governance literatures reinforces the existence of this structural gap. Digital governance research demonstrates that weak integration among technological, institutional, and social systems generates systemic vulnerabilities that propagate across governance ecosystems (Chernov, 2025). Studies of algorithmic governance similarly show that while AI can improve efficiency and transparency, it also introduces new forms of accountability uncertainty and governance complexity (de Gennaro et al., 2025). Across Sub-Saharan Africa, governments increasingly recognize the importance of AI governance but continue to face limited institutional capacity, fragmented regulatory frameworks, and underdeveloped data governance infrastructures (Ayana et al., 2024). Comparable conditions characterize fragile states such as Somalia, where digital transformation continues to expand despite persistent institutional constraints (Hashi, 2025).

Collectively, these limitations indicate that preserving trustworthy information ecosystems requires a complementary governance construct extending beyond conventional AI readiness. The gap is not merely technological. From an institutional perspective, formal rules may exist without sufficient legitimacy, coordination, or enforcement; from a policy-capacity perspective, governments may recognize a risk while lacking the analytical, operational, or political capability to respond; and from a socio-technical perspective, even sophisticated authentication tools can fail when they are disconnected from institutional procedures, legal authority, professional practice, and public interpretation (Scott, 2010; Wu et al., 2015; Sanfilippo et al., 2025). IIR therefore treats information integrity as a system-level governance capacity rather than a collection of isolated interventions (see Table 1).

**Table 1:** Conceptual boundaries between IIR and adjacent governance domains.

Domain	Primary object	Central question	Typical intervention	Boundary relative to IIR
AI readiness	AI adoption and governance	Can the state responsibly develop, deploy, and regulate AI?	Infrastructure, skills, strategy, ethics, regulation	Overlaps on institutions and regulation, but its primary object is AI systems and adoption rather than ecosystem-wide authenticity readiness.
Cybersecurity	Systems and information assets	Can systems resist unauthorized access, disruption, or compromise?	Security controls, incident response, resilience	Protects systems and information assets; synthetic content can cause harm without a technical compromise.
Misinformation governance	False or misleading content	How can harmful falsehoods be detected, moderated, or corrected?	Fact-checking, moderation, counter-messaging	Focuses on false or misleading content and response; usually not a multi-capacity national readiness architecture.
Media and information literacy	Individual interpretation	Can people critically evaluate information?	Education, awareness, verification skills	Contributes societal capacity but cannot substitute for institutional, technical, legal, and sector-specific readiness.
Digital resilience	Adaptation to digital risks	Can institutions and communities absorb and recover from digital disruption?	Preparedness, training, continuity, adaptation	Shares adaptation and resilience logic but is broader and not organized specifically around authenticity, provenance, and synthetic-media readiness.
Digital trust	Confidence in systems and institutions	Do users consider digital actors and services dependable?	Assurance, transparency, accountability	Trust is an outcome and relationship that IIR seeks to preserve; it is not itself a complete readiness architecture.
Synthetic-media governance	Creation, distribution, and harms of synthetic content	How should synthetic media be detected, disclosed, governed, and responded to?	Detection, labeling, provenance, platform rules, incident response	Closest substantive neighbor; typically organizes interventions or harms, whereas IIR organizes national preparedness across five interdependent capacities.
Information-Integrity Readiness	National information ecosystem	Can the state preserve authentic, trustworthy, and resilient information under pervasive synthetic media?	Coordinated institutional, technical, legal, societal, and sector-specific capacity	Integrates adjacent capacities around system-level preparedness; requires future operationalization and empirical validation.

The comparison clarifies that IIR does not claim novelty because its individual ingredients are entirely new. Its contribution is architectural: it changes the unit of analysis from an AI system, security incident, false claim, or individual user to the national information ecosystem; specifies five interdependent and partially non-substitutable readiness functions; and links those functions to maturity assessment and future operationalization. Adjacent domains can supply components of IIR, but the approaches reviewed here do not answer the same integrated readiness question.

## 4 Theoretical foundations of information-integrity readiness

The IIR framework is grounded in three complementary theoretical traditions. Their integration is necessary because synthetic-media governance simultaneously raises questions about institutional authority and legitimacy, the capacities required to formulate and implement policy, and the interaction between technical mechanisms and the social systems in which those mechanisms operate. Rather than assigning one theory to one pillar, the framework uses each theory as a cross-cutting explanatory lens across the five readiness dimensions.

### 4.1 Institutional theory: legitimacy, coordination, and regulatory capacity

Institutional theory explains how formal rules, organizational structures, norms, and shared expectations shape governance behavior and stability. Institutions are not effective merely because they formally exist; their authority also depends on legitimacy, accepted roles, and the capacity to coordinate behavior across organizational fields (Scott, 2010; Wang and Ching, 2013; Black, 2008). In information-integrity governance, this distinction is critical. A data-protection authority, cyber incident response team, media regulator, election body, or platform rule may exist formally while responsibilities remain fragmented, enforcement inconsistent, or public acceptance weak. Institutional readiness therefore concerns not only organizational presence but also recognized ownership, credible authority, interagency coordination, accountability, and the institutionalization of response procedures. This lens is especially relevant in fragile and developing digital states, where formal digital institutions may expand faster than the administrative routines and legitimacy required for coordinated implementation.

### 4.2 Policy capacity theory: analytical, operational, and political capability

Policy capacity theory provides a second mechanism for explaining variation in readiness. Wu et al. (2015) conceptualize policy capacity through analytical, operational, and political competencies operating across individual, organizational, and system levels. Analytical capacity concerns the ability to identify risks, interpret evidence, conduct forensic or policy analysis, and design proportionate interventions. Operational capacity concerns staffing, financing, interoperability, procedures, implementation, and the ability to translate formal commitments into action. Political capacity concerns legitimacy, stakeholder support, communication, coalition-building, and the ability to sustain policy action in contested environments (Wu et al., 2015; Howlett, 2016; Brenton et al., 2022). Applied to IIR, these capacities cut across all five pillars. Technical authenticity infrastructure requires analytical expertise and operational resources; legal safeguards require implementable rules and enforcement capacity; societal resilience depends partly on politically legitimate communication; and sector-specific readiness requires organizations capable of converting national policy into procedures. Policy failure may therefore result from imbalance among capacities even where formal institutions or technologies are present.

### 4.3 Socio-technical systems theory: interdependence and complementarity

Socio-technical systems theory emphasizes that technological and social arrangements are mutually dependent. In digital governance, outcomes emerge from interactions among technical infrastructure, organizational routines, regulation, professional practices, human interpretation, and wider institutional environments rather than from technology in isolation (Sanfilippo et al., 2025; de Gennaro et al., 2025). This mechanism is central to synthetic-media governance. Provenance metadata has limited value if platforms do not preserve it, institutions cannot interpret it, legal systems do not recognize it, journalists lack verification routines, or citizens do not understand authenticity signals. Conversely, strong media literacy cannot compensate for the absence of trusted verification infrastructure or enforceable accountability. IIR therefore conceptualizes the five pillars as complementary and partially non-substitutable components of one socio-technical governance system (Fuenfschilling and Truffer, 2014).

### 4.4 Integrated theoretical logic of IIR

Together, the three theories explain both the composition and the expected behavior of the IIR framework. Institutional theory explains whether governance arrangements possess legitimate authority, stable roles, and coordination; policy capacity theory explains whether actors possess the analytical, operational, and political capabilities needed to convert those arrangements into effective action; and socio-technical systems theory explains why effectiveness depends on alignment among institutions, technologies, laws, sectors, and societal users. The five pillars are therefore not simply thematic categories. They represent interdependent sites in which legitimacy, capacity, and socio-technical complementarity must be present if a state is to preserve information authenticity under conditions of pervasive synthetic media.

## 5 Developing the information-integrity readiness framework

Information-Integrity Readiness (IIR) is defined as the institutional, technical, legal and regulatory, societal, and sector-specific capacity of a state to preserve the authenticity, trustworthiness, and resilience of its information ecosystem in the era of synthetic media. Unlike conventional AI readiness, which evaluates preparedness to develop, deploy, and govern artificial intelligence technologies, IIR evaluates preparedness to protect the integrity of information generated, distributed, and consumed within AI-enabled environments. This distinction reflects an important evolution in governance priorities. As synthetic media increasingly blurs the boundary between authentic and fabricated communication, sustaining trustworthy information ecosystems becomes a governance objective rather than a secondary outcome of responsible AI regulation (Ferrara, 2026). The progressive erosion of shared epistemic foundations and declining confidence in institutional verification processes threaten democratic governance, public trust, and evidence-based decision-making (Ferrara, 2026; Romanishyn et al., 2025). Consequently, IIR conceptualizes information integrity as a multidimensional governance capacity requiring coordinated institutional, technical, legal, and societal preparedness.

Although IIR builds upon established governance scholarship, it represents a distinct conceptual domain rather than an extension of existing frameworks. AI governance primarily concerns policies, standards, regulations, and institutional oversight governing the development and deployment of AI systems (Saeed et al., 2023). Cybersecurity protects digital infrastructure and information assets from unauthorized access and technical compromise (Saeed et al., 2023). Media and information literacy strengthen individuals’ ability to critically interpret information within digitally mediated environments (Haider and Sundin, 2022), while digital resilience focuses on behavioural preparedness against misinformation and other digital risks (Soetekouw and Angelopoulos, 2024). Misinformation governance concentrates on countering false or misleading content, and digital trust examines confidence in institutions and information sources (Denniss and Lindberg, 2025; Romanishyn et al., 2025). IIR does not replace these governance domains. Instead, it integrates their complementary contributions into a unified readiness construct that evaluates whether countries possess the collective governance capacity required to preserve authentic information under conditions of pervasive synthetic media.

The framework emerged through the conceptual synthesis described in the Methodology rather than through the introduction of entirely new governance dimensions. Recurring concepts were grouped according to the governance function they perform in preserving information authenticity. Concepts concerning leadership, ownership, coordination, accountability, and incident response were clustered as institutional readiness; provenance, authentication, forensic capability, and verification as technical authenticity readiness; enforceable duties, platform obligations, remedies, evidentiary rules, and rights safeguards as legal and regulatory readiness; literacy, trusted communication, public awareness, and interpretive capability as societal resilience readiness; and domain-specific protocols, exercises, escalation thresholds, and continuity arrangements as sector-specific readiness. This functional clustering was then examined through institutional, policy-capacity, and socio-technical lenses to assess whether each pillar represented a distinct but interdependent governance function (Scott, 2010; Wu et al., 2015; Sanfilippo et al., 2025) (see Table 2).

**Table 2:** The five pillars of Information-Integrity Readiness.

Pillar	Core function	Governance focus	Illustrative future indicators
Institutional readiness	Coordinates governance across public and private actors	Ownership, accountability, and human oversight (Romanishyn et al., 2025).	Lead authority; interagency protocol; incident command; public communication mechanism; budget and workforce
Technical authenticity readiness	Establishes mechanisms for authentication, provenance, and verification	Cryptographic provenance and content transparency (Romanishyn et al., 2025).	Provenance standards; verification capacity; forensic capability; trusted identity integration; local-language tools
Legal and regulatory readiness	Creates enforceable accountability for synthetic-media harms	Transparency obligations and adaptive regulatory frameworks (Romanishyn et al., 2025).	Disclosure duties; platform obligations; remedies; evidentiary rules; rights safeguards; regulatory review
Societal resilience readiness	Strengthens public capacity to critically evaluate information	Digital literacy, cognitive resilience, and public awareness (Surjatmodjo et al., 2024)	Population awareness; journalist training; educational curricula; trusted channels; inclusion and accessibility
Sector-specific readiness	Adapts governance according to institutional context	Health, education, media, finance, and other critical sectors (Roe et al., 2024; Roppelt et al., 2024)	Sector protocols; risk assessments; exercises; escalation thresholds; continuity and recovery procedures

These pillars should not be interpreted as independent governance domains or as isolated policy interventions. Rather, they collectively represent the minimum governance capacities required to preserve information integrity within synthetic information environments. Weakness in any single pillar reduces the effectiveness of the others because information integrity depends on coordinated governance rather than isolated institutional performance. Institutional readiness provides strategic coordination and accountability; technical readiness enables provenance and verification; legal readiness creates enforceable responsibilities; societal readiness strengthens public resilience against manipulation; and sector-specific readiness ensures that governance mechanisms remain responsive to the operational realities of different sectors. Together, these dimensions shift governance attention from AI systems themselves toward the broader resilience of national information ecosystems.

Importantly, the framework conceptualizes readiness as an integrated governance system rather than a collection of independent capacities. Institutional leadership influences the implementation of technical provenance systems, while regulatory clarity determines whether organizations can operationalize authentication standards (Balasubramanian et al., 2021). Legal safeguards must simultaneously strengthen information integrity and protect fundamental rights, requiring complementary investments (Surjatmodjo et al., 2024). in public education, media literacy, and cross-sector collaboration (Surjatmodjo et al., 2024). Likewise, effective responses to synthetic media depend upon sustained cooperation among governments, academia, civil society, technology developers, journalists, and international partners (Tomassi et al., 2024). Information integrity is therefore best understood as a systems-governance challenge in which governance effectiveness emerges from interaction among multiple capacities rather than excellence within any single domain.

### 5.1 Conceptual propositions for future empirical testing

The propositions below are theory-derived expectations intended for future empirical testing; they are not findings established by this Perspective. They are stated to make the framework’s assumptions explicit and falsifiable. Institutional theory suggests that the object and legitimacy of governance shape the structures that states develop. Because IIR concerns the legitimacy and capacity of arrangements that preserve the evidentiary integrity of the information ecosystem, rather than primarily the development and regulation of AI systems, it should be empirically distinguishable from conventional AI readiness. Proposition 1 (future test): IIR will demonstrate conceptual and empirical distinctiveness from AI readiness because it assesses institutionalized capacity to preserve the integrity of the information ecosystem rather than capability to adopt and regulate AI systems.

Policy capacity theory predicts that governance performance depends on complementary analytical, operational, and political capabilities and that severe deficits in one capacity can undermine otherwise strong performance (Wu et al., 2015; Howlett, 2016). Applied to IIR, this yields a testable expectation rather than a demonstrated result. Proposition 2 (future test): Higher and more balanced capacity across the five IIR pillars will be associated with stronger national resilience to synthetic-media incidents than high performance in any single pillar alone.

Socio-technical systems theory provides the mechanism for the framework’s complementarity claim: technical mechanisms generate governance value only when institutions, rules, organizational routines, and users can interpret and act on them (Fuenfschilling and Truffer, 2014; Sanfilippo et al., 2025; de Gennaro et al., 2025). Proposition 3 (future test): The effect of technical authenticity mechanisms on information integrity will depend on institutional coordination, legal enforceability, and societal capacity to interpret and use verification signals.

Policy capacity must ultimately be translated from national arrangements into domain-specific operational routines. Because sectors differ in risk, evidence standards, professional practice, and consequences of failure, sector-specific readiness is treated as a potential mediating mechanism: national institutions and rules can affect frontline protection only when they are converted into sector protocols, escalation routines, verification procedures, and continuity arrangements. This mediating role is theoretical, not empirically established in the present Perspective. Proposition 4 (future test): Future empirical research should test whether sector-specific readiness mediates the translation of national governance capacity into operational protection in high-risk domains such as elections, health, finance, education, media, public administration, and national security.

### 5.2 From concept to measurement

Beyond providing a conceptual framework, IIR establishes a foundation for future operationalization. Each pillar can be translated into measurable indicators for comparative assessment, policy benchmarking, and national readiness evaluation. Consistent with policy capacity theory, measurement should distinguish formal capacity, implementation capacity, and demonstrated performance while also considering analytical, operational, and political capabilities (Wu et al., 2015). Formal capacity captures whether laws, institutions, standards, and plans exist. Implementation capacity captures staffing, funding, interoperability, training, coordination, and enforcement. Demonstrated performance captures whether institutions can detect, verify, communicate, and recover during realistic incidents. This approach avoids equating institutional presence with effective readiness.

A future IIR index should avoid the assumption that all dimensions are interchangeable. A high technical score should not compensate for the absence of legal remedies, and strong literacy programs should not compensate for weak incident coordination. A profile or dashboard approach may therefore be more informative than a single composite score. Where aggregation is used, minimum thresholds and imbalance penalties should be considered so that severe weakness in one pillar remains visible.

Operationalization should proceed through expert review, indicator specification, pilot testing, reliability assessment, construct validation, and comparative application. Mixed methods would be appropriate: document analysis for formal frameworks, administrative data for institutional resources, technical audits for provenance and forensic capability, population surveys for societal resilience, and sector exercises for operational performance. Although developing and validating such indicators lies beyond the scope of this Perspective, the framework provides the theoretical basis for future empirical measurement.

If supported empirically, stronger and more balanced Information-Integrity Readiness would be expected to reduce vulnerability to computational propaganda, strengthen democratic resilience, improve institutional trust, and enhance the long-term credibility of digital information ecosystems. These outcomes remain theoretical expectations for future validation. Because synthetic media exploits weaknesses across governance systems rather than within individual institutions, the framework predicts that effective preparedness depends on balanced capacity across all five readiness pillars.

## 6 Information-integrity readiness applied to Somalia’s digital landscape

Somalia provides an illustrative application of the Information-Integrity Readiness (IIR) framework because it combines rapid digital transformation with persistent institutional fragility. Expansion of telecommunications, mobile money, digital identity initiatives, and e-government have accelerated the country’s digital transition, creating new opportunities for public service delivery, financial inclusion, and citizen engagement (Hashi, 2025; Hassan, 2026). At the same time, institutional fragmentation, uneven digital infrastructure, limited regulatory capacity, and constrained governance resources continue to restrict the country’s ability to preserve trustworthy information ecosystems (Hashi, 2025). These conditions make Somalia an appropriate context for demonstrating why AI readiness and information-integrity readiness should be understood as complementary—but conceptually distinct—dimensions of national preparedness.

### 6.1 Qualitative maturity criteria for the illustrative assessment

Because the Somalia application is illustrative rather than a formal empirical index, readiness levels are assigned using transparent qualitative maturity criteria. Nascent indicates that relevant mechanisms are largely absent or *ad hoc*, with little evidence of institutionalized implementation. Emerging indicates that initial institutions, policies, initiatives, or capabilities exist but remain fragmented, weakly resourced, or inconsistently implemented. Developing indicates that formal structures and implementation mechanisms are present and expanding, although important gaps in coverage, coordination, enforcement, or resources remain. Established indicates that most core mechanisms are operational, coordinated, and supported by identifiable institutional capacity across relevant actors. Advanced indicates an integrated, routinely implemented, evaluated, adaptive, and sustainable readiness system with demonstrated performance. Each label is assigned by asking whether a relevant formal mechanism is documented, whether implementation is evidenced beyond formal existence, whether coordination and resources are visible, and whether routine operational use is demonstrated. Where the available secondary evidence does not document a mechanism, the assessment is treated as provisional rather than as proof of absence. These levels are interpretive descriptors, not statistical scores or national rankings.

Although Somalia has made important progress in strengthening its digital governance architecture, the distribution of readiness across governance capacities remains uneven. National digital identity initiatives—including e-Aqoonsi, HUBIYE, and the Certificate Delivery System—together with the 2025 Central Bank requirement for national identification in banking transactions, provide an important institutional foundation for trusted digital services (Isak and Mohamud, 2026). Similarly, the establishment of the Data Protection Authority, SOMCIRT, the Data Protection Act (2023) and the Cybersecurity Law (2026) demonstrate increasing recognition of digital governance priorities (Isak, 2026). These developments are treated here as enabling foundations rather than direct evidence of synthetic-media readiness. Dependence on foreign data hosting, limited AI-specific regulation, uneven implementation capacity, and the absence of dedicated synthetic-media governance continue to constrain the country’s ability to preserve information authenticity within increasingly AI-enabled information environments (Isak and Mohamud, 2026).

The analytical significance of Somalia is not simply that capacity is limited. It is that conventional AI readiness can register visible progress in connectivity, identity infrastructure, and digital services while overlooking whether institutions can establish provenance, coordinate authenticity disputes, communicate verified evidence, and manage synthetic-media incidents. Somalia therefore exposes the conceptual blind spot at the centre of the paper: digital adoption may advance faster than the capacity to preserve the evidentiary integrity on which digital governance depends.

Applying the proposed framework therefore illustrates unevenness rather than producing a deficiency ranking. The maturity labels are provisional diagnostic judgments tied to the criteria above and to the evidence summarized in Table 3; they do not represent a comprehensive legal or institutional audit. IIR is used to identify where documented governance foundations exist, where their implementation remains limited, and where synthetic-media-specific mechanisms are not yet evidenced.

**Table 3:** Illustrative assessment of Somalia through the five IIR pillars.

Pillar	Illustrative readiness	Evidence base and reason for label	Priority gap
Institutional	Emerging	SOMCIRT and the Data Protection Authority provide formal governance foundations but possess limited enforcement capacity (Isak and Mohamud, 2026). Relevant institutions therefore exist, but dedicated national ownership and routine cross-sector coordination for synthetic-media incidents are not yet demonstrated.	Clear national ownership, incident coordination, sustained resources, and cross-sector protocols
Technical	Emerging	National biometric identity systems provide trusted-identity foundations, although foreign data hosting constrains digital sovereignty (Isak and Mohamud, 2026). The cited evidence does not establish national provenance standards, synthetic-media forensic capacity, or interoperable authenticity infrastructure; this supports an Emerging rather than Developing classification.	Provenance infrastructure, local forensic capability, interoperability, and Somali-language verification tools
Legal and regulatory	Emerging	Data Protection Act (2023) and Cybersecurity Law (2026) provide initial legal foundations but lack AI- and synthetic-media-specific provisions (Isak and Mohamud, 2026). General digital-governance laws are present, but dedicated synthetic-media duties, remedies, and evidentiary rules are not yet evidenced.	Disclosure duties, platform accountability, remedies, evidentiary rules, and rights safeguards
Societal	Emerging	Digital literacy, institutional trust, and equitable digital access remain limited, particularly outside urban centres (Hassan, 2026; Isak and Mohamud, 2026). The cited evidence does not demonstrate institutionalized synthetic-media literacy or a routine trusted-verification communication system.	Public awareness, journalist capacity, trusted communication, and inclusion beyond urban populations
Sector-specific	Nascent	Governance mechanisms for synthetic media remain largely absent across health, elections, finance, and education (Isak and Mohamud, 2026). The cited evidence does not document sector-specific protocols, exercises, or escalation arrangements, supporting a Nascent classification.	Sector protocols, exercises, escalation thresholds, and continuity plans

The revised illustrative assessment identifies Emerging maturity for institutional, technical, legal and regulatory, and societal readiness, and Nascent maturity for sector-specific readiness. Foundational digital identity, cybersecurity, and data-protection developments justify movement beyond a purely nascent stage in several domains, but the available evidence does not establish the implementation depth required for a Developing classification in technical or legal readiness. These labels reflect the documented strength and institutionalization of available mechanisms, not a quantitative comparison with other countries. The weaknesses are also interconnected: limited digital literacy reduces the effectiveness of technical verification mechanisms; incomplete legal frameworks constrain institutional enforcement; and the absence of sector-specific governance limits the practical implementation of broader digital policies. From an IIR perspective, national preparedness therefore depends on balanced progress across all five pillars.

The value of the IIR framework becomes particularly evident when examining sector-specific vulnerabilities. Within healthcare, synthetic media and AI-generated health misinformation may influence treatment decisions, weaken confidence in trusted health authorities, and complicate public health communication (Denniss and Lindberg, 2025). During elections, synthetic media can manipulate political discourse, undermine electoral legitimacy, and erode confidence in democratic institutions (Porlezza, 2023). Financial systems increasingly rely upon trusted digital identities to reduce fraud associated with synthetic identities and AI-enabled deception (Isak and Mohamud, 2026; Porlezza, 2023). Within education, limited institutional preparedness constrains the responsible integration of AI-enabled technologies while increasing exposure to manipulated educational content (Isak and Mohamud, 2026). Public administration similarly faces the challenge of balancing digital innovation with transparency, cybersecurity, and institutional trust (Sharmin and Chowdhury, 2025). National security institutions confront parallel risks arising from AI-enabled hybrid threats and foreign information operations (Porlezza, 2023). Collectively, these examples demonstrate that information-integrity risks are systemic rather than sector-specific, reinforcing the need for a governance framework capable of coordinating preparedness across multiple institutional domains.

The Somali application also illustrates that Information-Integrity Readiness extends beyond evaluating current governance performance. Instead, it functions as a diagnostic framework capable of identifying structural governance imbalances before synthetic-media risks become institutional crises. By distinguishing between institutional, technical, legal, societal, and sector-specific capacities, the framework enables policymakers to prioritize investments according to governance needs rather than technological adoption alone. In resource-constrained settings, this distinction is particularly important because strengthening a single governance dimension is unlikely to produce sustainable information integrity without complementary progress across the remaining pillars.

Although Somalia serves as the illustrative application, the analytical value of IIR extends well beyond a single national context. Many fragile and developing digital states experience similar combinations of expanding digital transformation, uneven institutional capacity, evolving regulatory systems, and increasing exposure to synthetic media (Hashi, 2025). Evidence from developing countries consistently demonstrates that digital inclusion, institutional capability, cybersecurity, and digital literacy jointly influence the effectiveness of digital governance and sustainable development (Atobishi and Mansur, 2025). Likewise, the rapid evolution of generative AI continues to outpace conventional regulatory approaches, reinforcing the need for adaptive, participatory, and forward-looking governance (Taeihagh, 2021; Walter, 2024). Investments in synthetic-media detection, information authentication, digital literacy, and collaborative AI governance should therefore be understood as complementary components of national information-integrity readiness rather than isolated policy interventions (Capraro et al., 2024).

## 7 Illustrative transferability example: Kenya

Kenya is included only as a transferability illustration, not as a second case study or a basis for country performance comparison. It is used to ask whether the same five-pillar architecture can organize information-integrity issues in a different African digital-governance context. Kenya represents a developing digital state with a more mature AI-policy ecosystem, including a national AI strategy and an evolving legislative debate, yet it continues to confront information-integrity risks associated with deepfakes, misinformation, crisis communication, digital literacy, and the translation of general AI governance into operational safeguards (Amatika, 2022; Karanja and Nzau, 2026; Onunga, 2026). The section therefore does not assign Kenya maturity labels, compare national performance, or support causal claims.

Viewed through the IIR framework, Kenya’s experience illustrates how the same diagnostic categories can be applied without assuming the same institutional trajectory as Somalia. Institutional and policy development can strengthen governance foundations, but synthetic-media resilience additionally requires authentication and forensic capacity, enforceable rules for harmful synthetic content, trusted public communication, and sector-specific response procedures. Research on deepfake regulation in Kenya has already emphasized the need for forensic expertise and public awareness, while recent crisis-communication research identifies deepfakes as an emerging challenge in environments characterized by misinformation and uneven digital literacy (Amatika, 2022; Karanja and Nzau, 2026). These concerns map onto the technical, legal, societal, and sector-specific pillars of IIR; the purpose here is conceptual mapping, not scoring.

The Kenya illustration supports only a limited portability claim: IIR can serve as a common diagnostic architecture across distinct settings when its indicators, thresholds, and evidence requirements are adapted to national context. It does not establish external validity, comparative performance, or universal causal effects. Those claims require future empirical comparative research. The conceptual contribution of IIR, the illustrative Somalia application, and the Kenya transferability example should therefore be interpreted separately from conclusions that depend on future validation.

## 8 Policy and practice recommendations

Strengthening Information-Integrity Readiness (IIR) requires coordinated investments across institutional, technical, legal, societal, and sector-specific governance capacities rather than isolated efforts to regulate artificial intelligence. The theoretical synthesis suggests three implementation principles: governance arrangements must possess legitimate and clearly recognized institutional authority; analytical, operational, and political capacities must be developed in combination; and technical interventions must be aligned with the social, legal, and organizational systems through which they are used (Scott, 2010; Wu et al., 2015; Sanfilippo et al., 2025). For fragile and developing digital states, governance should therefore prioritize scalable, context-sensitive interventions that strengthen the authenticity, trustworthiness, and resilience of national information ecosystems.

### 8.1 Establish institutional ownership and incident coordination

Governments should establish a designated national coordination mechanism with a clear mandate for synthetic-media preparedness and response. It should connect public institutions, academia, civil society, media organizations, and the private sector while adopting risk-based governance approaches proportionate to available institutional capacity (Hashi, 2025; Romanishyn et al., 2025). Core functions should include threat assessment, escalation protocols, public communication, cross-border cooperation, and post-incident learning.

### 8.2 Build authenticity and provenance infrastructure

Priority should be given to developing provenance infrastructure, authenticity verification mechanisms, and Somali-language AI tools capable of identifying manipulated content while ensuring interoperability, privacy protection, and equitable access (Bengio et al., 2024; Waditwar, 2025). Resource-constrained states should emphasize shared verification services, partnerships with universities and media organizations, and procurement rules that require content transparency and auditable provenance.

### 8.3 Modernize legal and regulatory safeguards

Legal and regulatory frameworks should evolve beyond conventional cybersecurity and data protection legislation by incorporating adaptive approaches that improve transparency, strengthen platform accountability, and address AI-generated content while safeguarding fundamental rights and freedom of expression (Gregory, 2022; Romanishyn et al., 2025). Laws should distinguish malicious deception from legitimate expression, define disclosure and record-keeping duties, establish remedies for identity and reputational harm, and clarify how synthetic evidence is treated in judicial and administrative processes.

### 8.4 Strengthen societal resilience and trusted communication

Governments should invest in sustained digital literacy, synthetic-media awareness, journalist capacity-building, and public education delivered through partnerships among educational institutions, civil society, technology developers, and the media (Islam et al., 2026). Programs should move beyond generic awareness by teaching citizens how provenance signals work, when verification is necessary, which institutions are trusted to confirm evidence, and how uncertainty should be communicated during rapidly evolving incidents.

### 8.5 Translate national capacity into sector-specific protocols

Sector-specific implementation should recognize that synthetic media presents different governance challenges across healthcare, elections, finance, education, public administration, and national security. Governments should therefore promote interdisciplinary research, encourage platform accountability, and strengthen international cooperation through regulatory harmonization, technical assistance, and culturally appropriate AI solutions that reinforce information integrity across critical sectors (Hashi, 2025; Romanishyn et al., 2025). Each critical sector should establish risk scenarios, escalation thresholds, verification procedures, public communication templates, and continuity arrangements.

### 8.6 Adopt a phased implementation roadmap

Implementation should be phased according to national capacity. Short-term priorities include assigning institutional ownership, strengthening digital literacy, developing Somali-language AI verification tools, and establishing risk-based governance mechanisms. Medium-term priorities should focus on institutional capacity building, provenance infrastructure, legal modernization, and cross-sector coordination. Long-term priorities should emphasize adaptive regulation, regional and international cooperation, recurring preparedness exercises, and sustainable governance systems capable of responding to future synthetic-media threats (Romanishyn et al., 2025) (see Figure 1).

**Figure 1:**
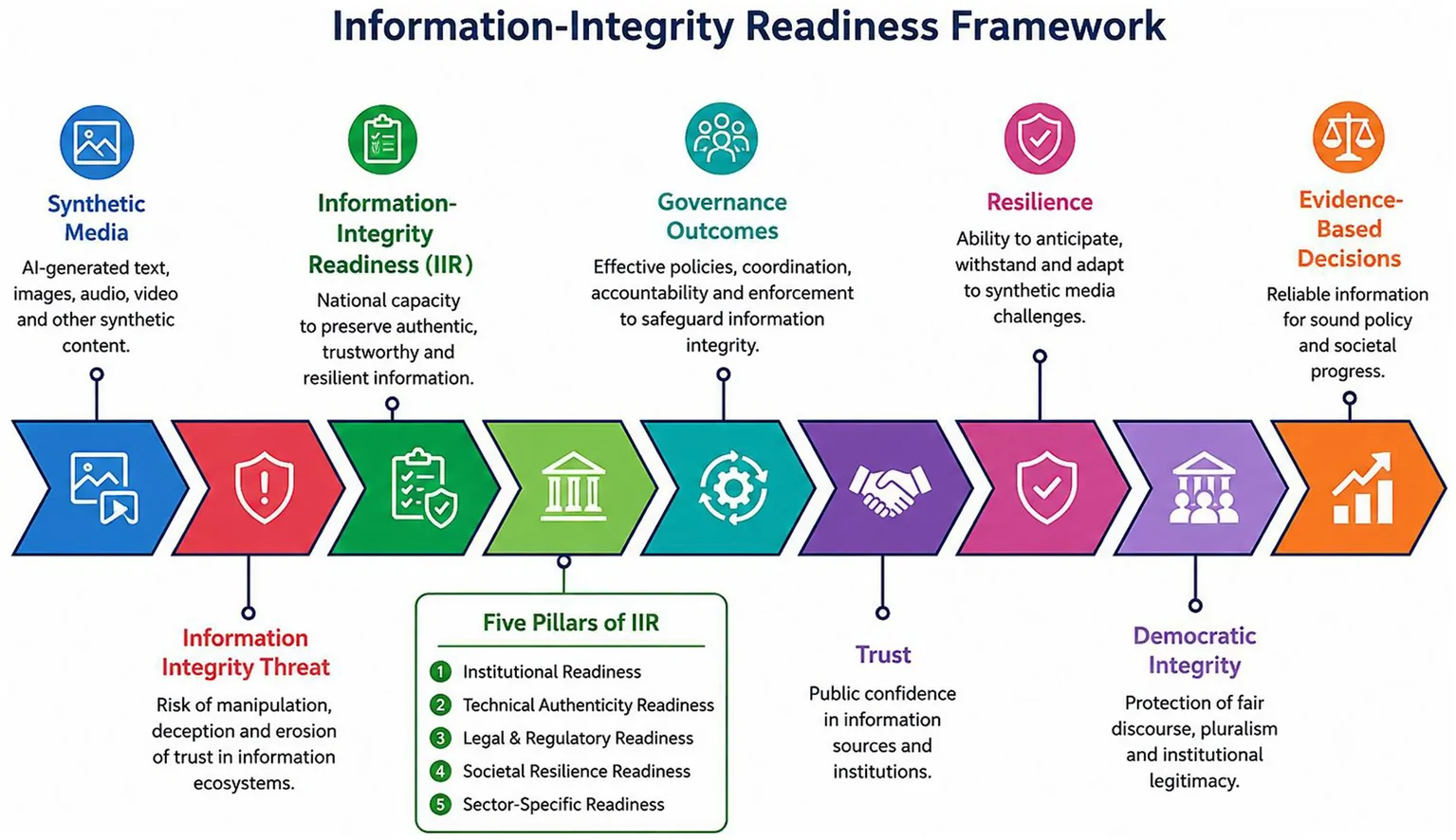
Information-integrity readiness framework for governing synthetic media. IIR, information integrity readiness.

The figure summarizes the causal logic of the Perspective: synthetic media creates an information-integrity threat; the Information-Integrity Gap becomes visible when conventional readiness frameworks do not assess this threat; IIR organizes the required capacities through five interdependent pillars; and balanced readiness supports governance outcomes expressed through trust, resilience, democratic integrity, and evidence-based decision-making.

## 9 Research agenda and limitations

Future research should first operationalize the five pillars into observable indicators and test whether the construct exhibits convergent, discriminant, and predictive validity. Comparative studies should examine whether IIR explains variation in synthetic-media resilience beyond conventional AI readiness, cybersecurity maturity, institutional quality, and digital literacy. Sector-level studies should test how national capacity is translated into operational performance in elections, health, finance, education, media, public administration, and national security.

A second research priority is theoretical validation. Comparative and longitudinal studies should test whether institutional legitimacy and coordination explain variation in IIR implementation, whether analytical, operational, and political policy capacities predict differences across pillars, and whether socio-technical alignment moderates the effectiveness of technical authenticity mechanisms. These tests would move the framework from theory-informed conceptual development toward explanatory and predictive validation.

A third priority is to evaluate interaction and threshold effects. The framework predicts that pillars are interdependent and partially non-substitutable. Empirical work should therefore test whether technical capability produces limited benefits when legal authority, institutional coordination, operational capacity, or societal understanding is weak. Simulation exercises, synthetic-media incident studies, expert elicitation, and longitudinal policy analysis could provide useful evidence.

This Perspective has limitations. The framework is derived from narrative conceptual synthesis rather than a systematic review or empirical validation. The five pillars organize recurring governance capacities but do not claim to exhaust all country-specific determinants of information integrity. The Somalia application is illustrative and relies on available secondary evidence; the revised maturity labels are provisional evidence-based judgments, not statistically derived scores, a comprehensive primary-source audit, or a formal national ranking. The Kenya section is a transferability illustration rather than a substantive comparative case and therefore does not establish cross-country performance differences or external validity. The four propositions are theory-derived expectations for future testing, including the proposed mediating role of sector-specific readiness; none is presented as an empirically established finding. The framework also does not prescribe a universal weighting scheme because the relative salience of pillars may vary across political, institutional, and sectoral contexts. These boundaries distinguish the conceptual contribution of IIR from its illustrative country applications and from conclusions that require future empirical validation.

## 10 Conclusion

The rapid evolution of generative AI has transformed information integrity into a fundamental governance challenge that extends beyond conventional AI readiness. While existing readiness frameworks effectively assess AI adoption and governance, they provide limited guidance for evaluating national capacity to preserve authentic and trustworthy information ecosystems. This Perspective addresses that gap by introducing Information-Integrity Readiness (IIR) as a complementary governance framework for assessing preparedness against synthetic-media threats.

The revised framework is theoretically anchored in institutional theory, policy capacity theory, and socio-technical systems theory. These perspectives explain, respectively, why legitimate authority and coordination matter, why analytical, operational, and political capacities must be balanced, and why technical authentication cannot be separated from institutions, laws, professional practices, sectors, and public interpretation. The five IIR pillars therefore represent theoretically connected and partially non-substitutable governance capacities rather than a descriptive checklist.

Using Somalia as an illustrative application, supported by transparent qualitative maturity criteria and explicit evidence-to-label reasoning, the framework illustrates how rapid digital transformation can coexist with uneven information-integrity capacity. The Kenya section provides a limited transferability illustration showing that the same diagnostic architecture can organize information-integrity issues in a different institutional setting; it is not a comparative country assessment. These applications do not constitute empirical rankings or causal tests. Future research should operationalize the pillars through measurable indicators, test the proposed theoretical mechanisms and propositions—including the potential mediating role of sector-specific readiness—and undertake comparative validation. The next phase of AI governance should therefore move beyond asking whether states are ready to adopt and regulate artificial intelligence. It must also ask whether they possess the legitimate institutions, policy capacities, socio-technical arrangements, and sectoral procedures required to preserve the authenticity of the information on which public trust, democratic integrity, institutional legitimacy, and evidence-based decisions depend.

## Data Availability

The original contributions presented in the study are included in the article/supplementary material, further inquiries can be directed to the corresponding author.
